# Relationship between the stiffness of the medial gastrocnemius and spatiotemporal variables during 100-m sprint running in collegiate sprinters

**DOI:** 10.1371/journal.pone.0347567

**Published:** 2026-04-30

**Authors:** Norifumi Fujii, Katsuki Takahashi, Raki Kawama, Taku Wakahara

**Affiliations:** 1 Graduate School of Health and Sports Science, Doshisha University, Kyoto, Japan; 2 Organization for Research Initiatives and Development, Doshisha University, Kyoto, Japan; 3 Faculty of Health and Sports Science, Doshisha University, Kyoto, Japan; 4 Faculty of Sport Sciences, Waseda University, Saitama, Japan; 5 Human Performance Laboratory, Waseda University, Saitama, Japan; Erzurum Technical University: Erzurum Teknik Universitesi, TÜRKIYE

## Abstract

Higher stiffness of the medial gastrocnemius (MG) has been reported to be associated with faster race time in 100-m sprint running. However, the association of muscle stiffness with spatiotemporal variables (i.e., step frequency, step length, flight time, stance time, flight distance, and stance distance), which are determinants of sprint running velocity, remains unknown. Thus, we aimed to elucidate the relationship between the passive/active stiffness of MG and spatiotemporal variables at the 50–60 m interval (maximal velocity phase) during 100-m sprint running. Using an ultrasound scanner (11 Hz), the shear wave velocity of MG in 21 male collegiate sprinters (100-m best race time: 11.03 ± 0.29 s) was measured at passive/active (during 20% and 50% of maximal voluntary isometric contraction [MVC] of plantar flexion) states as a proxy for muscle stiffness. Participants performed 100-m sprint runs, and their sprint running motion at the 50–60 m interval was recorded using a camera (240 Hz) to calculate spatiotemporal variables. The results showed that the shear wave velocity of MG at neither passive (0% MVC) nor active (20% and 50% MVC) states correlated with 100-m sprint time (*r* = 0.189, 0.331, 0.102, *p* = 0.620, 0.429, and 0.659, respectively) or any spatiotemporal variables (*r* = −0.264 to 0.422, *p* = 0.137 to 0.937). These results suggest the stiffness of MG might not be important for spatiotemporal variables of sprinters, at least with the performance level in this study.

## Introduction

Sprint running, the fastest form of locomotion over the ground for humans, is required in many sports. Sprint running performance is represented by running velocity, which is determined by spatiotemporal variables (i.e., step frequency, step length, flight time, stance time, flight distance, and stance distance) [1–3]. A previous study reported that the step frequency was positively correlated with running velocity among the students participating in sprint-based sports [1]. The step frequency is determined by flight time and stance time [2]. On the contrary, another study reported that step length was positively correlated with running velocity among athletes participating in sprint-based sports [2]. The step length is determined by flight distance and stance distance [2]. Meanwhile, distinct strategies of spatiotemporal variables were observed in national- or international-level sprinters; some achieved high running velocity through high step frequency, while others achieved it through long step length [3]. Therefore, high running velocity is achieved by various spatiotemporal variables in highly trained sprinters. These spatiotemporal variables during sprint running have been reported to be affected by several factors, such as muscle size [4,5] and the timing of muscle activities [6,7]. Elucidating the factors affecting spatiotemporal variables and sprint running performance will provide valuable insights for athletes and coaches in establishing effective training programs for enhancing sprint running performance.

Recent studies [8,9] have reported that sprint running performance is related to muscle stiffness, assessed as shear modulus with ultrasound shear wave elastography (SWE) in well-trained sprinters. For example, Miyamoto et al. (2019) reported that the stiffness of the vastus lateralis at the passive state was negatively correlated with 100-m race time, but that at the active (during isometric contractions at 50% of maximal voluntary contractions [MVC]) state was positively correlated with 100-m race time [8]. In another study [9], the stiffness of the medial gastrocnemius (MG) at the passive and active (during isometric contractions at 20%, 50%, and 80% MVC) states was negatively correlated with 100-m race time. Thus, muscle stiffness may be a determinant of 100-m sprint running performance. It should be noticed here that sprint running performance is determined by spatiotemporal variables. Nevertheless, no studies have investigated the relationships between passive/active muscle stiffness and spatiotemporal variables during sprint running.

During sprint running, MG contracts in a stretch-shortening cycle (SSC) pattern [10]. The SSC is an action of the muscle-tendon unit that involves an active stretch (eccentric contraction) followed by immediate shortening (concentric contraction). The MG also contracts in the SSC pattern during drop jump, which is a typical SSC exercise in the vertical direction [11]. Ando et al. (2021) reported that drop jump height, an indicator of SSC performance, was positively correlated with the passive stiffness of MG [12]. Thus, the passive stiffness of MG may also be related to the horizontal SSC performance (i.e., sprint running).

The purpose of this study was to elucidate the relationships between passive/active MG stiffness and spatiotemporal variables during 100-m sprint running in collegiate sprint runners. It was hypothesized that stiffer MG at the passive and active states would be positively correlated with longer flight distance during sprint running, which is a determinant of step length. This study could provide valuable information for understanding the mechanisms of how muscle stiffness plays a role in sprint running.

## Materials and methods

### Participants

Twenty-one male collegiate sprint runners (age: 20.9 ± 1.2 years, body height: 173.6 ± 3.6 cm, body mass: 65.3 ± 4.4 kg) participated in this study. An *a priori* power analysis was performed using statistical software (G*Power version 3.1.9.7, Heinrich Heine University, Germany) to find statistically significant correlations between passive/active stiffness of MG and sprint running performances, including the spatiotemporal variables. A two-tailed correlation test with an alpha level of 0.05 and a power of 80% was used in this calculation. The effect size was assumed to be 0.583 based on the previous study (at passive state, [9]). As a result, the sample size was estimated to be 20, and thus 21 male sprint runners were recruited for this study.

Seventeen out of 21 participants specialized in sprint running events (100 m, 200 m, and 400 m), and the rest of the participants specialized in jumping (long jump) or combined (decathlon) events. The inclusion criteria were (i) male sprint runners belonged to university track and field club, (ii) had 100-m best race time faster than 11.70 s, (ⅲ) participation in at least a 100-m race during the season of the year when this experiment was conducted, (ⅳ) at least 3 years of competitive experience in sprint running, and (ⅴ) had no musculoskeletal injuries of the lower limbs when the experiment was conducted, which would prevent them from performing MVC or sprint runs at maximal effort. The exclusion criterion was sprint runners who got injured in their lower limbs during the measurement period. Their personal best time for the official 100-m race ranged from 10.63 to 11.56 s (11.03 ± 0.29 s), and their experience in sprint running was 9.1 ± 2.5 years. Participants were recruited between August 8 and September 1, 2024. After an explanation of the purpose, procedures, burdens, and risks of this study, written informed consent was obtained from all participants. This study was approved by the Doshisha University Research Ethics Review Committee (21035−2).

### SWE measurement

The posture of the participants and experimental procedure for SWE measurement were based on the previous study [9]. Participants lay prone on an experimental bed with their hips at 0° and their knees fully extended. Their right foot was securely attached to a footplate of a dynamometer (Biodex System 4, Biodex Medical System, USA) with non-elastic straps. The rotational axis of their right ankle joint was carefully aligned with that of the footplate of the dynamometer. The ankle joint angle was set at 10° plantar flexion (0° = anatomically neutral position).

The shear wave velocity map of MG was acquired using an ultrasound scanner (Aixplorer Ver.8, Supersonic Imagine, France) with a linear array probe (2–10 MHz, SL 10−2, Vermon, France) in the SWE mode (MSK preset, persistence = off, smoothing = 5). The ultrasound probe was manually placed at 30% of the lower leg length (from the popliteal crease to the lateral malleolus) and 40% of the MG width (from the boundary between MG and flexor digitorum longus to that between MG and lateral gastrocnemius). The fascicle direction of MG was identified on the B-mode image, and the ultrasound probe was aligned with the direction. At the passive state, three images of the MG shear wave velocity map were recorded while the participants were instructed to completely relax. After a warm-up exercise consisting of five submaximal voluntary isometric contractions of plantar flexion, they performed two MVCs of plantar flexion for 3 s. A rest period of 3 min was provided between the two MVCs. The torque signal was measured with the dynamometer at a sampling frequency of 2 kHz using an analog-to-digital converter (PowerLab 16/35, ADInstruments, Australia). The higher value of MVC torque was used to set the target torques of 20% and 50% MVC. The target torques were decided based on the previous study [9]. Although the previous study [9] included 80% MVC as a target torque level, we excluded this contraction intensity due to the difficulty of obtaining reliable measurements in our preliminary experiments. The target torque was displayed as a guideline on a computer screen for real-time visual feedback. The participants performed two submaximal plantar flexions (approximately 7 s) at each intensity for SWE measurements. During the plantar flexions, a series of MG shear wave velocity maps over 7 s was recorded as a video clip at a sampling rate of 11 Hz. The order of 20% and 50% MVC was randomized across participants. A rest period was provided for 3 min between different contraction intensities and for 2 min between trials.

**Fig 1:**
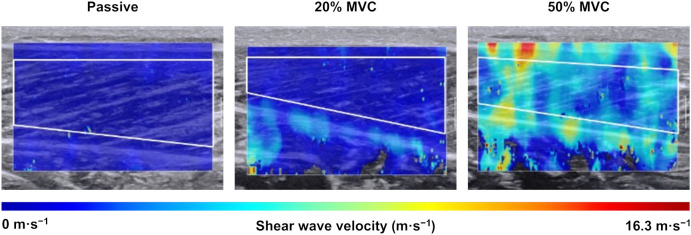
Typical examples of ultrasound shear wave elastographic images of the medial gastrocnemius at the passive and active (20% and 50% maximal voluntary contraction [MVC]) states, respectively. The colored region represents the shear wave velocity map with the scale beside the images. The solid white line indicates the area selected as the region of interest.

The analysis of recorded images was performed using the software built into the ultrasound scanner (Q-box Trace, Supersonic Imagine, France). Three frames were selected from the video in each state (passive, 20% and 50% MVC). Stable color areas within the MG in the shear wave velocity map were manually traced as the region of interest (ROI), avoiding the saturated areas, missing areas, aponeurosis, and subcutaneous adipose tissues with reference to the B-mode images (Fig 1). The ROI was set as large as possible, ensuring that no pixels within the ROI exceeded the saturation limit of the shear wave velocity (< 16.3 m·s^−1^). The spatial average of shear wave velocity was calculated in the ROI. The shear wave velocities across three images in one trial were averaged in the passive state. In the active state, the average value of the six data (three images for each of two trials) was used for further analysis. The mean coefficients of variation of the shear wave velocities at the passive and active (20% and 50% MVC) states were 1.9 ± 1.4%, 5.1 ± 6.7%, and 3.8 ± 3.3%, respectively. The intraclass correlation coefficients at the passive and active (20% and 50% MVC) states were ≥ 0.880, ≥ 0.770, and ≥ 0.661, respectively.

During the SWE measurement, electromyographic (EMG) activity of the MG was recorded using a wireless EMG system (Trigno Wireless System, Delsys, USA). The EMG electrode was placed near the ultrasound probe after skin preparation, including shaving, abrasion with sandpaper, and cleaning with alcohol wipes to decrease skin impedance. The EMG and torque signals were synchronized using recording software (LabChart v8.1.18, ADInstruments, Australia). In the EMG data of the MVC trial, the root-mean square (RMS) value was calculated 1 s around (0.5 s before and after) the instance of the peak torque. The RMS-EMG value during SWE measurements was calculated for 1 s during the passive state and for the last 5 s of the submaximal plantar flexion task in the active states. The RMS-EMG values during SWE measurements were normalized to those during the MVC trial.

### Sprint running measurement

After 60 min of warm-up, including jogging, dynamic stretching, and short sprint running, participants performed two 100-m sprint runs. They ran on a synthetic track using their usual sprint running spikes and clothing. A rest period of 40 min was provided between the two trials based on previous studies [13,14]. The sprint running measurements were conducted on three separate days for all the participants and not implemented on days with low temperatures (less than 20°C) or rain. The average temperature for 3 hours during the 3 days of the measurement was 25.6 ± 2.2°C. The weather conditions at the measurement time were referred to data published by the Japan Meteorological Agency.

**Fig 2:**
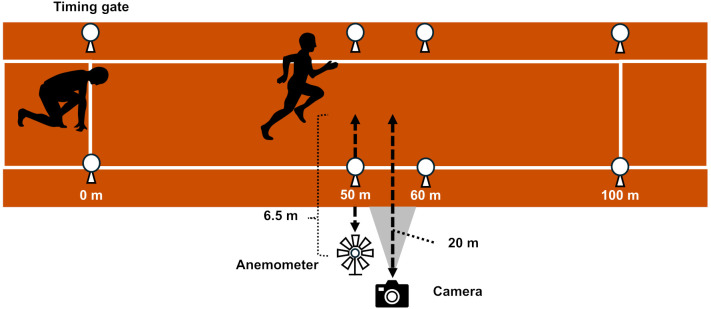
The experimental settings of sprint running measurement.


Measurement procedures and device settings were based on a previous study ([13], Fig 2). Four pairs of timing gates (Witty Gate, Microgate, Italy) were placed at 0, 50, 60, and 100 m from the starting point to measure sprint running times of 50–60 m and 0–100 m intervals. We defined the 50–60 m interval as the maximal velocity phase and measured the sprint running time of this interval according to a previous study [15]. The height of the timing gates was set at 1 m from the ground to match the height of the hip joints of the participants. To capture a video of the 50–60 m interval and calculate the spatiotemporal variables, a camera (HAS-U2, DETECT, Japan, frame rate: 250 fps, exposure time: 1/3000 s) was placed at 55 m from the starting point. The camera was placed 20 m apart from the middle of the running lane. To record the wind velocity, a digital anemometer (866B Pro Anemometer, Kethvoz, China) was placed 6.5 m apart from the middle of the running lane at 50 m. Participants started sprint running at their arbitrary timing after the wind velocity was less than ± 2.0 m·s^−1^. The wind effects on the 100-m sprint running time were accounted for by using the following equation [16]:

$$T_{corrected}=T-\text{0}.\text{0}\text{4}\text{4}\text{9}w+\text{0}.\text{0}\text{0}\text{9}\text{4}\text{5}\text{9}Tw-\text{0}.\text{0}\text{0}\text{4}\text{2}w^{\text{2}}$$

where $T_{corrected}$ means 100-m sprint running time (s) corrected for wind magnitude (m·s^−1^), *T* means recorded 100-m sprint running time (s), and *w* means wind magnitude (m·s^−1^).

The faster 100-m sprint run was used for further analysis. The calculation procedure for spatiotemporal variables was similar to the previous study [5]. The mean sprint running velocities of 50–60 m and 0–100 m intervals were calculated by dividing the interval distance (10 m and 100 m) by the sprint running times of each interval. Image analyzing software (ImageJ, National Institute of Health, USA) was used to calculate spatiotemporal variables for the right leg. The stance and flight phases were defined as the timing from the right foot touch down to the right toe off and from the right toe off to the left foot touch down, respectively. The stance and flight times were calculated by dividing the number of frames in the stance and flight phases by the frame rate of the camera (250 fps). The step time was defined as the sum of the stance and flight times, and the inverse of the step time was defined as the step frequency. The stance and flight distances were defined as the distances that the anterior-posterior center of the greater trochanter moved horizontally during the stance and flight phases, respectively. The step length was defined as the sum of the stance and flight distances. The normalized step frequency was calculated by multiplying the step frequency by the square root of body height [17]. The normalized step length was calculated by dividing the step length by body height [17]. These spatiotemporal variables were calculated twice for the first step on the right leg from 50 m, and the mean of the two values was used for further analysis.

### Statistics

The Shapiro-Wilk test was used to evaluate the distribution of the shear wave velocity at each contraction intensity and the sprint running performance variables (*T*_*corrected*_ and spatiotemporal variables). As a result, all data did not violate the assumption of normality (*p* = 0.074 to 0.929). Hence, parametric tests were used for the statistical analyses of this study. Pearson’s product-moment correlation coefficient was used to investigate the relationships between two measured variables. The significance level was set at *p* < 0.05. The Benjamini-Hochberg method was used to correct the *p* values based on the number of the target torque levels (passive, 20%, and 50% MVC) at a false discovery rate of < 0.05. Linear multiple regression tests were also performed with each spatiotemporal variable as the dependent variable and the shear wave velocity of MG under three conditions (passive, 20%, and 50% of maximal voluntary isometric plantar flexion) as the independent variables. Statistical analyses were performed using statistical software (IBM SPSS Statistics Ver. 29, IBM, USA).

## Results

The MVC torque of plantar flexion was 83.6 ± 13.6 Nm. The exerted torque during the SWE measurement at 20% and 50% MVC corresponded to 24 ± 1% and 53 ± 2% of MVC, respectively. When measuring the shear wave velocity of passive MG, the RMS-EMG value of MG was 2.0 ± 1.5% of that during MVC. The average shear wave velocities were 3.0 ± 0.2 m·s^−1^ (passive), 5.9 ± 1.2 m·s^−1^ (20% MVC), and 8.2 ± 1.0 m·s^−1^ (50% MVC), respectively.

**Fig 3:**
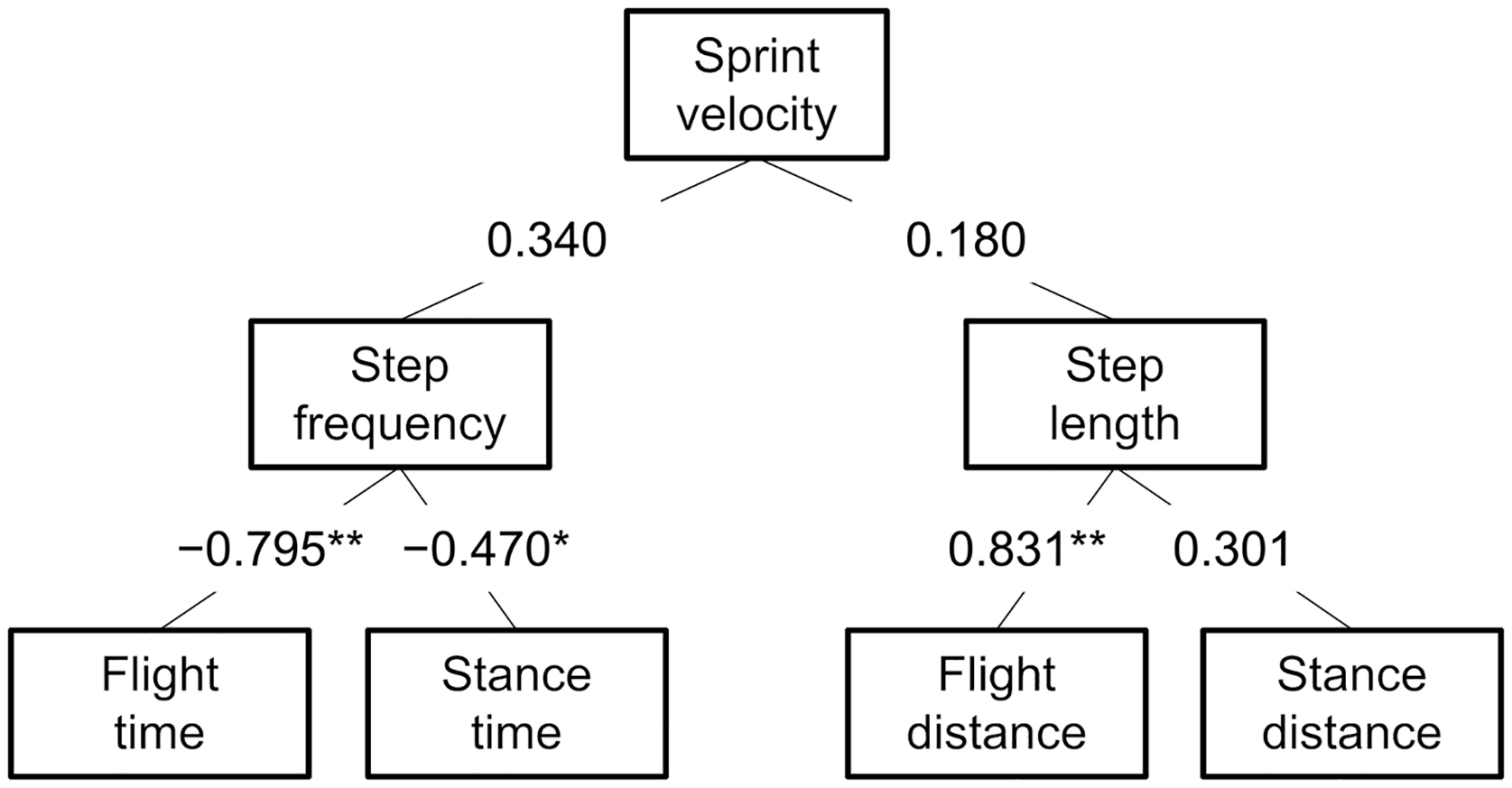
Correlation coefficients between the sprint running velocity and the spatiotemporal variables at 50–60 m intervals of 100-m sprint running for 21 participants. * Indicates a significant correlation (*p < 0.05, **p < 0.01).

The average wind velocities in the 3 days of measurements were −0.2 ± 0.7 m·s^−1^ at the time of the start of the 100-m sprint running. The *T*, *T*_*corrected*_, and sprint running time of 50–60 m intervals were 11.32 ± 0.29 s, 11.30 ± 0.29 s, and 1.02 ± 0.04 s, respectively. The sprint running velocities of 0–100 m (calculated using *T*_*corrected*_) and 50–60 m intervals were 8.85 ± 0.23 m·s^−1^ and 9.83 ± 0.37 m·s^−1^, respectively. Correlations between the sprint running velocity and the spatiotemporal variables at 50–60 m intervals are presented in Fig 3. Sprint running velocity was not significantly correlated with step frequency (*r* = 0.340, *p* = 0.131) or step length (*r* = 0.180, *p* = 0.434). Step frequency was significantly correlated with flight time (*r* = −0.795, *p* < 0.001) and stance time (*r* = −0.470, *p* = 0.031). The step length was significantly correlated with flight distance (*r* = 0.831, *p* < 0.001), but not with stance distance (*r* = 0.301, *p* = 0.184).

**Fig 4:**
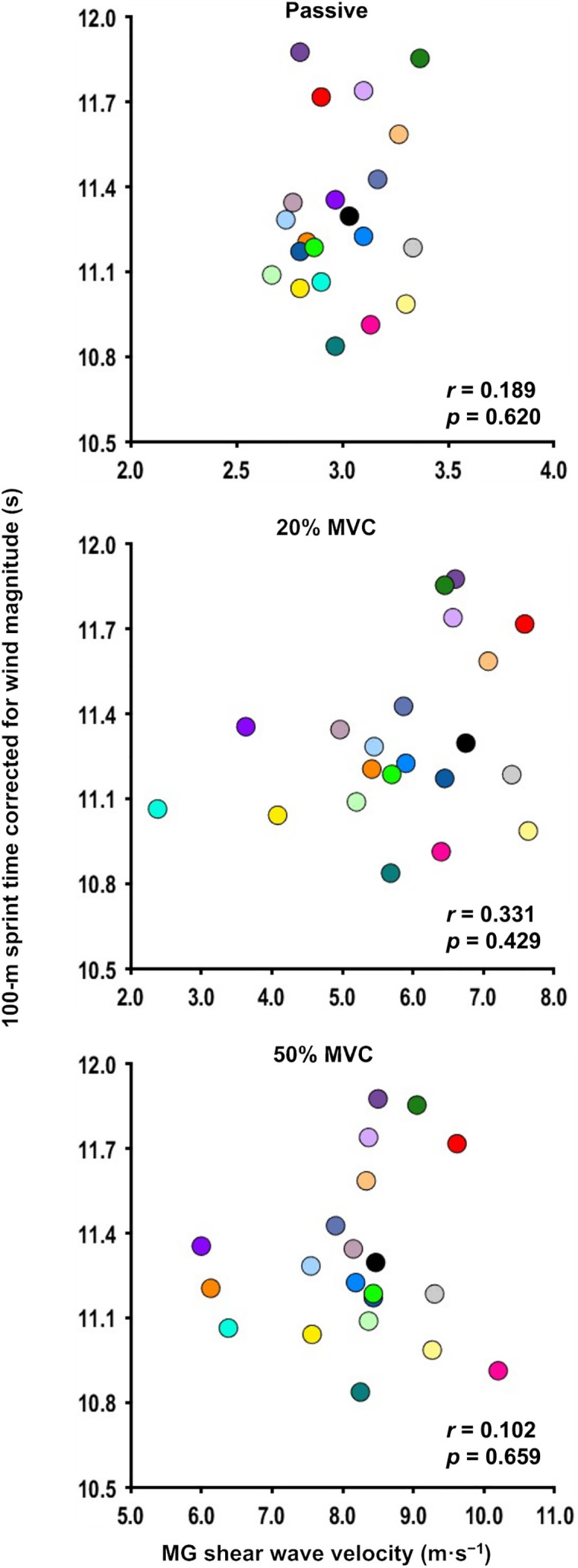
Relationship between shear wave velocity of the medial gastrocnemius at the passive/active (20% and 50% maximal voluntary contraction [MVC]) states and the 100-m sprint time. The 100-m sprint time was corrected for wind.

Fig 4 shows the scatter plots of shear wave velocity under the passive/active states and *T*_*corrected*_. No significant correlations existed between *T*_*corrected*_ and the shear wave velocity under the passive state (*r* = 0.189, *p* = 0.620), 20% MVC (*r* = 0.331, *p* = 0.429), or 50% MVC (*r* = 0.102, *p* = 0.659).

**Fig 5:**
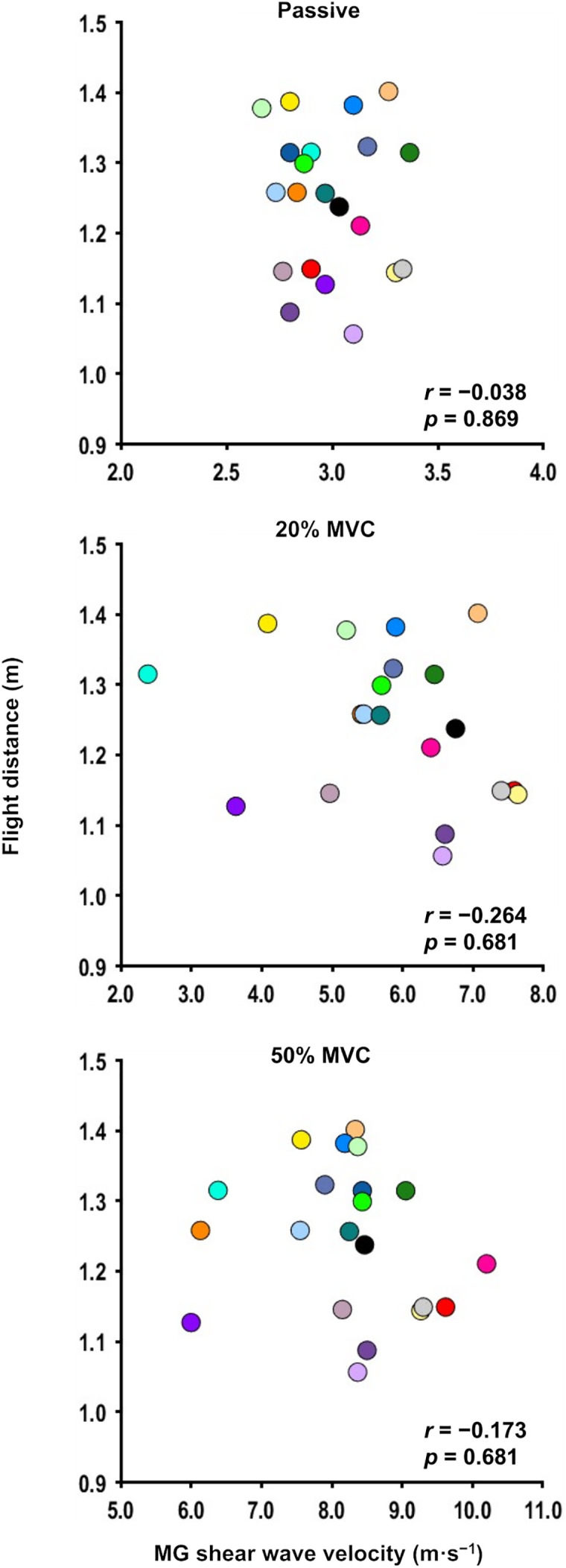
Relationship between shear wave velocity of the medial gastrocnemius at the passive/active (20% and 50% maximal voluntary contraction [MVC]) states and flight distance.

Fig 5 shows the relation between shear wave velocity under the passive/active states and flight distance. No significant correlations were found between flight distance and the shear wave velocity under the passive state (*r* = −0.038, *p* = 0.869), 20% MVC (*r* = −0.264, *p* = 0.681), or 50% MVC (*r* = −0.173, *p* = 0.681).

Table 1 shows the correlation coefficients between the shear wave velocity under passive/active states and spatiotemporal variables. There were no significant correlations between the measurement variables (*r* = −0.264 to 0.422, *p* = 0.137 to 0.937). In addition, no shear wave velocities of MG were selected as a significant predictor for the spatiotemporal variables (*β* = −0.482 to 0.459, *R*^2^ = −0.163 to 0.051, *F* = 0.066 to 1.356, *p* = 0.221 to 0.995).

**Table 1:** Relationships between the MG shear wave velocity and spatiotemporal variables.

Spatiotemporal variables	MG shear wave velocity					
	**Passive**		**20% MVC**		**50% MVC**	
***r***	***p***	***r***	***p***	***r***	***p***
Step frequency	−0.089	0.762	−0.070	0.762	−0.088	0.762
Normalized step frequency	−0.149	0.722	−0.099	0.722	−0.082	0.722
Step length	−0.039	0.868	−0.224	0.868	−0.053	0.868
Normalized step length	0.045	0.868	−0.113	0.868	−0.039	0.868
Stance time	0.117	0.614	0.422	0.137	0.379	0.137
Flight time	0.063	0.786	−0.198	0.770	−0.151	0.770
Step time	0.128	0.721	0.083	0.721	0.098	0.721
Stance distance	0.018	0.937	0.073	0.937	0.238	0.894

MG: medial gastrocnemius, MVC: maximal voluntary contraction.

The individual results of the shear wave velocity of the MG and spatiotemporal variables during sprint running are shown in the supplementary information file (S1 Table).

## Discussion

The present study showed that the shear wave velocity of MG at passive and active states did not correlate with any spatiotemporal variables or *T*_*corrected*_ in male collegiate sprinters (Table 1). The results of the present study do not support the hypothesis that stiffer MG at the passive and active states would be positively correlated with longer flight distance during sprint running. It has been shown that the MG stiffness is correlated with 100-m race time in sprint runners [9], but its relationship with spatiotemporal variables remains unknown. The present results suggest that MG stiffness has a trivial effect on sprint running time and spatiotemporal variables in collegiate sprinters.

The lack of correlation between the shear wave velocity of MG and *T*_*corrected*_ was inconsistent with previous results [9], which may be caused by several factors. Because the sample size (*n* = 21) of the present study was calculated based on the previous result (*n* = 18, *r* = −0.583, *p* = 0.011 [passive state], [9]), the lack of correlation is unlikely to be explained by our sample size. On the contrary, the inconsistent results may be associated with the differences in the performance levels of the participants and/or the measurement method of the 100-m sprint time. The best records of 100-m sprint running were 11.03 ± 0.29 s (mean ± SD, from 10.63 to 11.56 s) in the present study and 10.88 ± 0.33 s (from 10.25 to 11.38 s) in the previous study [9]. In the previous study [9], three participants with the best records of ≤ 10.50 s exhibited especially higher stiffness of MG at all active states (> 7.0 m·s^−1^ at 20% MVC, > 9.5 m·s^−1^ at 50% MVC, and > 13.0 m·s^−1^ at 80% MVC), while such fast runners were not included in the present study. The correlations between the stiffness of MG and 100-m race time may be attributed to the data of such fast runners in the previous study [9]. Meanwhile, *T* was measured during the maximal effort trial with correction for wind velocity in the present study. Our analysis did not include the reaction time at the start of the 100-m sprint running. On the other hand, the previous study [9] used the official 100-m race time within 1.5 months before and after the experimental day (3 months in total) for analysis. The official 100-m race time should have been influenced by the wind velocity and reaction time. These methodological differences between the present and previous studies may also affect the discrepancy in the correlation between MG stiffness and sprint running time.

The sprint velocity at the 50–60 m interval was not correlated with step frequency or step length in this study (Fig 3). As sprint velocity is the product of step frequency and step length, these results indicate that the sprint performance of our participants did not depend on either step frequency or step length. In other words, some sprinters were fast because of their high step frequency, while others were also fast because of their long step length. Our result is inconsistent with the previous studies [1,2], which reported a significant correlation between sprint velocity and step frequency/step length. This inconsistency suggests that the sprint runners of the present study may be atypical ones. We recruited well-trained collegiate sprint runners (100-m best race time: 10.63–11.56 s). On the contrary, previous studies [1,2] recruited physical education students, including several sprinters (experimental 100-m sprint time: 10.35–15.03 s [1]) and athletes whose sports involved sprint running (100-m best race time was not reported [2]). Therefore, the atypicality of the spatiotemporal variables in our participants might be due to the homogeneous performance level of our participants. Another possible factor of the atypical correlation is the heterogeneity of step frequency/step length. A previous study [18] reported the significant correlation between the sprint velocity and step frequency in the participants with homogeneous step length (2.07–2.15 m), and conversely, between sprint velocity and step length in participants with homogeneous step frequency (4.51–4.72 Hz). Meanwhile, our participants exhibited both wide ranges of step frequency (4.17–5.00 Hz) and step length (1.97–2.38 m). Simultaneously representing a wide range of step frequency and step length may affect the correlation between sprint velocity and step frequency/step length. Taken together, the atypicality of the spatiotemporal variables in the present participants may affect the correlation between the MG stiffness and step frequency/step length. Furthermore, to consider the effect of body height on step frequency and step length, normalized values were used in the analysis. Nevertheless, normalized step frequency and normalized step length did not correlate with passive or active MG stiffness. Thus, body height may have a small effect on the correlation between MG stiffness and step frequency/step length.

Contrary to our hypothesis, no correlations were found between the shear wave velocity of MG at passive and active states and flight distance (Fig 5). Meanwhile, the previous study [12] reported that the passive stiffness of MG was positively correlated with drop jump height. A possible factor contributing to the discrepant findings is ankle joint kinetics during the stance (ground contact) phase. The stance time of sprint running (84–108 ms) was shorter than the ground contact time during the drop jump (148–290 ms [12]). Meanwhile, the ankle plantar flexion torque during the stance phase did not differ substantially between sprint running (2.60 ± 0.28 Nm·kg^−1^ [19]) and drop jump from 30 cm height (2.21 ± 0.42 Nm·kg^−1^ [20]). Thus, the ankle plantar flexion power during the stance phase of sprint running (29.06 ± 4.15 W·kg^−1^ [19]) was more than twice as large as that during the drop jump from 30 cm height (11.05 ± 2.86 W·kg^−1^ [20]). Although we did not examine the ankle joint kinetics, these mechanical differences between sprint running and drop jump may affect the relationship between drop jump height/flight distance and MG stiffness.

### Limitations and future directions

This study has several limitations. Although we used the higher value of the two trials, the MVC torque in seven out of 21 participants increased by more than 5% from the first to the second trial. We did not conduct another trial or extend the trial duration for those participants. However, they might have achieved a higher torque in another trial or in a longer-duration trial, and the relative intensity of plantar flexion during SWE measurements might have been incorrect if such participants were present. Therefore, we could not rule out the possibility that the lack of correlation between the shear wave velocity of MG and spatiotemporal variables resulted from the insufficient control of relative intensities of plantar flexion during SWE measurements across participants.

The active stiffness of the MG was measured during submaximal voluntary contraction at 20% and 50% MVC. In contrast, sprint running is performed with maximal effort. A previous study [21] reported a linear correlation between shear modulus of the abductor digiti minimi and isometric contraction intensity (0–100% MVC). If this finding extends to MG, the stiffness of MG even at a higher contraction intensity would not correlate with *T*_*corrected*_. In addition, the MG stiffness was measured only in one specific region (30% of the lower leg length and 40% of the MG width) and at a specific ankle joint angle (10° of plantar flexion) based on previous studies [9,12]. Meanwhile, the MG stiffness was reported to be varied along the regions [22] and with the ankle joint angle [23]. Thus, the relationships between MG stiffness and spatiotemporal variables may differ depending on the measurement region within the muscle and ankle joint angle during the measurement. Therefore, future studies are warranted to examine the correlations between MG stiffness at multiple regions/ankle joint angles and spatiotemporal variables.

## Conclusion

This study aimed to elucidate the relationships between passive and active stiffness of MG and spatiotemporal variables during 100-m sprint running in collegiate sprinters. The present results showed that the passive and active stiffness of MG did not correlate with 100-m sprint running time or spatiotemporal variables at 50–60 m intervals. These results suggest that the MG stiffness at passive and active states might not be important for the spatiotemporal variables of the sprinters with a 100-m sprint running time of around 11 s.

## Supporting information

S1 Table Shear wave velocity of the medial gastrocnemius, sprint running time, sprint running velocity, and spatiotemporal variables in each participant. (XLSX)
